# CeleryDB: a genomic database for celery

**DOI:** 10.1093/database/bay070

**Published:** 2018-07-09

**Authors:** Kai Feng, Xi-Lin Hou, Meng-Yao Li, Qian Jiang, Zhi-Sheng Xu, Jie-Xia Liu, Ai-Sheng Xiong

**Affiliations:** State Key Laboratory of Crop Genetics and Germplasm Enhancement, Key Laboratory of Biology and Germplasm Enhancement of Horticultural Crops in East China, Ministry of Agriculture, College of Horticulture, Nanjing Agricultural University, Nanjing 210095, China

## Abstract

Celery (*Apium graveolens* L.) is a plant belonging to the Apiaceae family, and a popular vegetable worldwide because of its abundant nutrients and various medical functions. Although extensive genetic and molecular biological studies have been conducted on celery, its genomic data remain unclear. Given the significance of celery and the growing demand for its genomic data, the whole genome of ‘Q2-JN11’ celery (a highly inbred line obtained by artificial selfing of ‘Jinnan Shiqin’) was sequenced using HiSeq 2000 sequencing technology. For the convenience of researchers to study celery, an online database of the whole-genome sequences of celery, CeleryDB, was constructed. The sequences of the whole genome, nucleotide sequences of the predicted genes and amino acid sequences of the predicted proteins are available online on CeleryDB. Home, BLAST, Genome Browser, Transcription Factor and Download interfaces composed of the organizational structure of CeleryDB. Users can search the celery genomic data by using two user-friendly query tools: basic local alignment search tool and Genome Browser. In the future, CeleryDB will be constantly updated to satisfy the needs of celery researchers worldwide.

Database URL: http://apiaceae.njau.edu.cn/celerydb

## Introduction

Celery (*Apium graveolens* L.) is a plant belonging to the Apiaceae family originated from the Middle East and the Mediterranean, and is one of the most important vegetables worldwide (1). Celery is widely cultivated owing to its low calorie count and abundant celluloses, vitamins and carotenes. Previous studies have found that celery possesses numerous medicinal functions, such as inhibiting cancer cell growth and decreasing blood pressure (2, 3). Celery cultivated in China is mainly classified into two groups, namely, Chinese celery (also known as local celery) and Western celery (introduced from Western countries). The ‘Q2-JN11’, a local celery, is a highly inbred line obtained by artificial selfing of ‘Jinnan Shiqin’. Physiological and molecular investigations are necessary to address the increasing demand for celery.

With the development of sequencing technology and molecular biology, many genetic researches on celery were reported. The transcriptome sequences of *A. graveolens* cv. ‘Ventura’ leaves at different stages were *de novo* assembled, and the results provided useful information on lignin accumulation in celery (4). Fu et al. (5) recognized several molecular markers of celery by transcriptome sequencing. Simple sequence repeat markers and differentially expressed genes were identified from two celery cultivars using deep transcriptome sequencing (6). Comparative proteomic analysis was conducted on celery to understand the defense system under temperature stresses (7). High-throughput sequencing of small RNAs of celery varieties identified the abiotic stress-related microRNAs (8, 9). The application of next-generation sequencing (NGS) technology to plants has provided considerable information on the genetic resources for researchers worldwide (10). However, to our knowledge, a public genomic database of celery is currently unavailable. On the basis of the whole-genome sequences of *A. graveolens* cv. ‘Q2-JN11’, we constructed CeleryDB (http://apiaceae.njau.edu.cn/celerydb), a genomic database for celery.

The *de novo* assembled whole-genome sequences of *A. graveolens* cv. ‘Q2-JN11’ are available on CeleryDB. The CeleryDB website consists of five interfaces, namely, Home, BLAST, Genome Browser, transcription factor (TF) and Download. The collective data presented in this database is expected to provide valuable resources for genetic and genomic studies on celery.

## Data resources

### Plant materials

The data presented in the current version of CeleryDB was derived from the genome sequence of Q2-JN11 celery (a highly inbred line obtained by artificial selfing of ‘Jinnan Shiqin’). Celery seeds were deposited and sowed at the State Key Laboratory of Crop Genetics and Germplasm Enhancement, Nanjing Agricultural University. The celery seedling was grown under the condition of 12-h light at 22°C and 12-h dark at 18°C, with a relative humidity of 60–70%. The whole genome sequences, nucleotide sequences of predicted genes and amino acid sequences of predicted proteins are available on CeleryDB.

### Genome sequencing and assembly

The genomic DNA was extracted from the young leaves of ‘Q2-JN11’ celery using CTAB method with some modifications (11). The genomic DNA was sequenced using the HiSeq 2000 platform (BGI-Shengzhen). Raw data were filtered for adaptor contamination and low quality by using CutAdapt prior to assembly. Then, the cleaned data were assembled using SOAPdenovo2 software (http://soap.genomics.org.cn/soapdenovo.html) (12). Finally, we obtained an assembly of 3.18 Gb.

### Gene prediction and annotation

A total of 34 277 putative genes were predicted using Augustus 3.2.2 software and SNAP program (13, 14). The average length of putative genes and numbers of exons per putative genes was 3267 bp and 5.27, respectively. The predicted genes were annotated based on the alignments to the NCBI non-redundant protein sequence, Swiss-Prot, TrEMBL and gene ontology (GO) databases with BLASTp at *E* values of 1 × 10^−4^ (15, 16). The GO IDs for the predicted genes were obtained using Blast2GO (17).

### Identification and classification of TF

**Figure 1.**
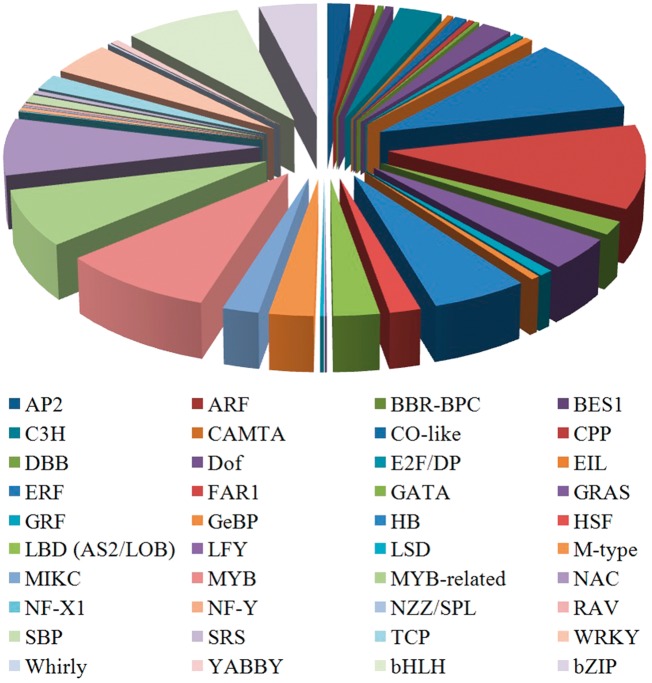
Distribution of various TF families in celery.

TFs bind to special sites of the target gene to regulate the gene transcription during plant biological processes (18, 19). TFs can be grouped into various families on the basis of the DNA-binding domains of the protein sequences (20). The sequences of other known TFs were downloaded from PlnTFDB (21). The conserved domains of various TF families were used as queries to search against the predicted celery proteins to obtain the celery TFs. Prediction of TFs in the CeleryDB database was accomplished using HMMER software (22). In the celery genome, a total of 1698 TFs that were classified under 40 families were identified and provided (Figure 1). Among the 40 families, the FAR1 family showed the largest number of TF members, followed by the ERF and MYB TF families. The numbers of TFs in the FAR1, ERF and MYB families were 185, 161 and 154, respectively.

## Database construction

### Implementation of CeleryDB

**Figure 2.**
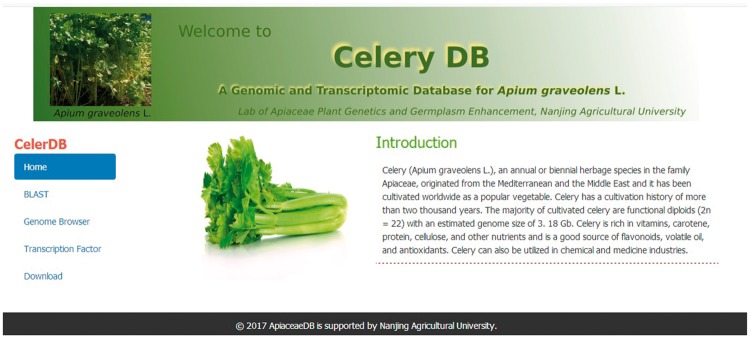
Homepage of CeleryDB.

To make the genomic data of celery available, the user-friendly website database CeleryDB was developed and constructed. We used the Linux (CentOS6.2) system as the server, Apache HTTP as the web server and PHP5 for Web development. Perl scripts, HTML and JavaScript were also used to build the website. The basic local alignment search tool (BLAST) and Genome Browser services were installed in this website database (23). CeleryDB allows users to access BLAST and browse the celery genome data, and to download the sequences of putative genes and proteins. The CeleryDB website consists of five interfaces, namely, Home, BLAST, Genome Browser, TF and Download. The Home interface provided an introduction and images of celery (Figure 2). Here, users can acquire the basic information on celery.

### Basic local alignment search tool

**Figure 3.**
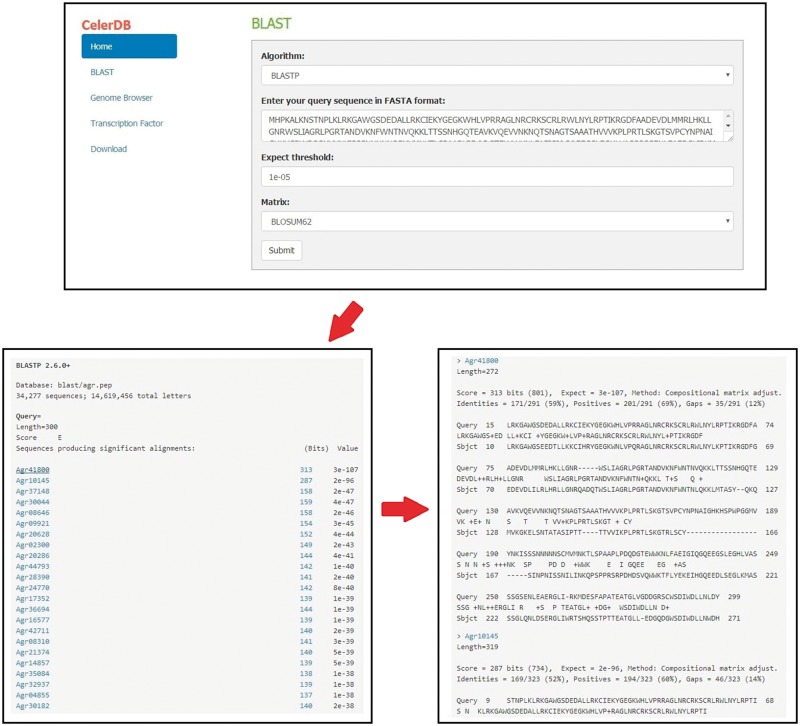
Detailed procedures for BLAST search on CeleryDB.

The BLAST program was embedded in the CeleryDB Web interface to allow users to perform sequence alignment (24). Users can obtain target genes from the celery database on the basis of sequence similarity by using BLAST program. The sequences of the whole genome, putative genes, and putative proteins of celery are available through the BLAST program. Prior to BLAST, users should enter the query sequence in FASTA format, select the algorithm (BLASTp or BLASTx), and set the associated parameters (Expect threshold and Matrix) (Figure 3). Then, clicking the Submit icon allows users to go to the query result interface. The IDs corresponding to the query sequence of celery are listed and ordered based on the alignment scores. Clicking the Bits icon behind the gene IDs provides the link to the alignment results interface.

### Genome browser

**Figure 4.**
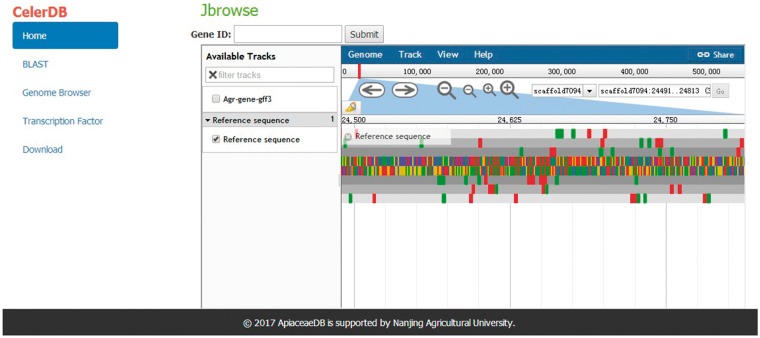
Interface of Genome Browser on CeleryDB.

For exhibiting the annotation of the celery genome, a Genome Browser that well integrates the database and interactive web pages was embedded in CeleryDB (23). The Genome Browser allowed users to track the annotations of genes, mRNA, coding sequence (CDS) and transcripts of each scaffold. The various annotations of the scaffolds were marked with different icons in this browser. Users can acquire the detailed features of various annotations by clicking the corresponding icons (Figure 4).

### Transcription factor

**Figure 5.**
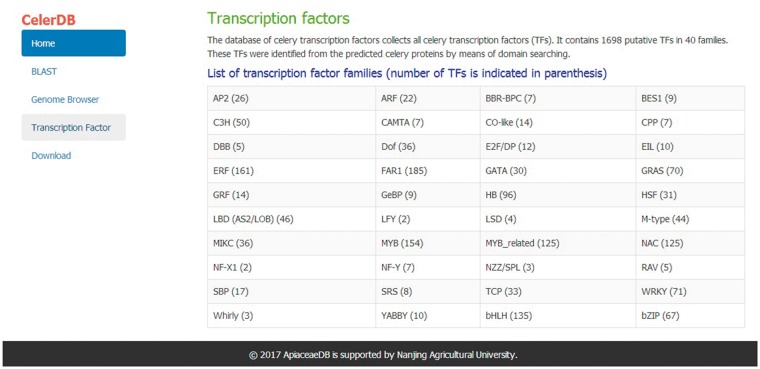
Number of different TF families and the interface of TF.

TFs are vital regulators with highly conserved DNA-binding domains during plant growth development and stress response (18, 25). Previous studies demonstrated that numerous known TFs play significant roles in various biological processes. For example, the TFs of Dof and TCP families are involved in plant growth and development; some TFs of the NAC family are related to stress response; and numerous TFs of the MYB family function in the biosynthesis of secondary flavonoid metabolites (26–29). For convenience, the 1698 celery TFs belonging to 40 families are listed in a table on the TF interface (Figure 5). The numbers of different TF families were indicated in parenthesis. Users can download the TF data in the Download section.

### Download

**Figure 6.**
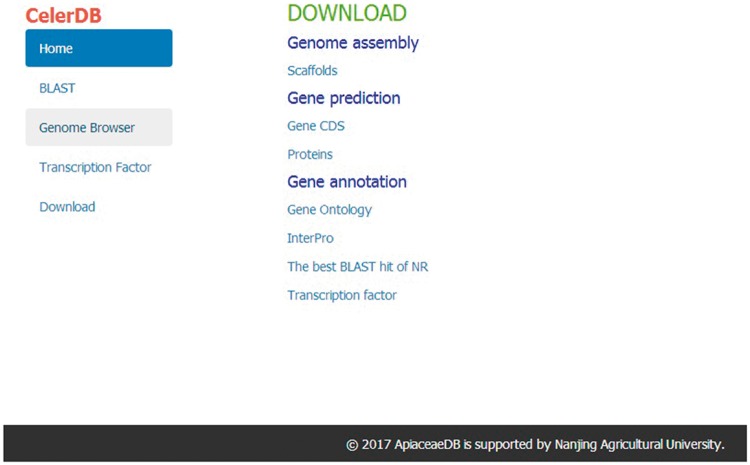
Interface of Download on CeleryDB.

The Download interface allows users to freely download the celery genomic data for further analysis. The available data included the scaffolds of the genome assembly, the CDS of predicted genes, the amino acid sequences of predicted proteins and the gene annotation (GO, InterPro, The best BLAST hit of NR and TF) of the celery genome (Figure 6).

### Allergenic protein genes in celery

Celery is recognized as a healthy vegetable because of its abundant nutrients over the world, whereas celery is also one of the common plant food sources that cause allergic reactions in central European human (30–32). The allergenic reaction to celery induces many symptoms such as human oral allergy, severe cases exhibited life-threatening anaphylactic reactions (33, 34). So far, several allergens were identified from celery, including Api g 1 (35), Api g 2 (36), Api g 4 (37), Api g 5 (38) and Api g 6 (39). Numerous studies indicated that Api g 1 is the most important allergen to human in celery (40, 41). To examine the quality of CeleryDB database, we used the sequence of known Api g 1 (GenBank accession No. Z75662.2) as query to search against the celery genome to obtain the allergenic protein gene. Search results indicated that Agr42308 in CeleryDB was the most similar to the sequence of Api g 1, with similarity of 100%. Agr42308 sequence contained a 480 bp open reading frame that encoded 159 amino acids. The above retrieved sequence in CeleryDB will be useful in future studies of celery allergens. These results indicated that the data in CeleryDB is efficient and accurate for celery genome research.

### Discussion and future plans

Celery is a popular vegetable worldwide because of its abundant nutrients and various medicinal effects (1). Despite the numerous genetic and molecular biology studies on celery, no public database of the celery genome is currently available worldwide. In view of the significance of celery and the development of bioinformatics, an online database based on the genome of ‘Q2-JN11’ celery named CeleryDB was constructed. To our knowledge, CeleryDB is the first public genome database with functional annotations for celery. The sequences of the whole genome, putative genes and putative proteins of celery are available on CeleryDB. In addition, CeleryDB identifies and provides the putative TFs from the whole genome sequence of celery. To enable users to obtain the celery genomic data, we embedded two user-friendly query tools, namely, BLAST and Genome Browser, into CeleryDB.

CeleryDB was targeted to be a flexible computational platform for future genetic studies on celery. With the development of sequencing technology and the increasing studies on celery, various celery transcriptomes will be published in the coming years. Therefore, CeleryDB will be constantly updated with new information to satisfy the increasing research demands. We hope our efforts will make CeleryDB a helpful database for celery genome research.

## Funding

The research was supported by the New Century Excellent Talents in University (NCET-11-0670); National Natural Science Foundation of China (31272175); Jiangsu Natural Science Foundation (BK20130027); Priority Academic Program Development of Jiangsu Higher Education Institutions (PAPD).

*Conflict of interest*. None declared.
